# Efficacy of Electricity in Dental Surgery—Scirrhus and Epithelial Degeneration of the Tongue and Tissues at Its Base

**Published:** 1877-12

**Authors:** David Prince


AMERICAN JOURNAL

OF

DENTAL SCIENCE.

Vol. XI. THIRD SERIES—DECEMBER, 1877. No. 8.

### ARTICLE I.

### *Efficacy of Electricity in Dental Surgery—Scirrhus and Epithelial Degeneration of the Tongue and Tissues at its Base.*

[MESSRS. EDITORS:—Among the numerous reports tending to sustain my position as to the Efficacy of Electricity in Dental Surgery, I give you the subjoining as one of interest, especially as it comes from DAVID PRINCE, M. D., a physician of large experience and practice.

Yours fraternally,

NEW YORK, Nov. 2nd, 1876. GEO. H. PERINE.]

Scirrhus and Epithelial degeneration of the tongue, and of the tissues at its base, present unusual difficulties to the application of the knife, the scissors, and the ligature.

The difficulty of access, and the abundant vascularity, all points to the desirability of some agent which will sever the parts without loss of blood, and without the necessity of ligatures.

The vessels are in the midst of muscular fibers crossing in various directions on account of which a tenaculum is liable to tear out, and unless a pin is inserted previously to the application of a ligature it is liable to slip off. A deluge of blood in the mouth obscures the parts upon which the knife or scissors are being applied. The cleansing away of blood retards the progress of the operation, so that the surgeon is forced to proceed with uncertainty in regard to the precise place of the deeper incisions. The Galvano-cautery meets these difficulties; there need be no blood lost, though the prick of the tenaculum or vulsellum employed to hold the tongue is apt to occasion the loss of a few drops. It is not that the loss of a small quantity of blood in an operation is any serious evil to a patient if it can be securely checked; it is the great satisfaction to the surgeon in being able to proceed by one step, or by many, with accuracy and with the parts in perfect view that gives this proceeding its attractive features and entitles it to be called beautiful. For great accuracy of detail and perfection of result, it is best to proceed by short steps, or as we should say in the use of the knife by short incisions, time is in this case of very little consequence in comparison with perfection of result. It is convenient to reverse the order of incision by the scalpel and to imitate that of the bistoury, *i. e.*, to cut from within outward. The platinum wire is introduced by a needle threaded by the wire; or better by a thread which serves as a pilot for the wire. Having been introduced and attached to the wire looop-holder the connection with the battery is made, and the wire slowly cuts through, closing vessels as it proceeds. In operating upon a part so vascular as the tongue, it is best to use a wire as large as No. 1 of the French and American scales for bougies, because it coagulates deeper than a small wire, which lacks the necessary radiation of heat to destroy more than a very small thickness of substance. In parts presenting very little vascularity, a small wire can be used with rapid progress, but with the risk of melting at some point. The inconvenience arising from this accident, however, is only that of delay in re-adjusting the wire. Surgery in its esthetic aspect always loses credit by mishaps producing delay, and on this account it is desirable to work with appliances which do not get out of order in the act of using them. In the removal of the lateral half of the tongue, it is convenient to split the tongue first. To this end a thread is introduced at the point intended to be the posterior extremity of the incision ; this thread acts as a pilot for the platinum wire, which when drawn in is attached to the loop-holder, having connection with the battery. At this stage the median line of the tongue is embraced in a loop of wire.

The writer finds it convenient to control the current by pedal, the strength of the current and the consequent degree of heat are determined by the depth to which the plates sink in the fluid. All things being in readiness, the plates are immersed by an assistant, while the operator makes traction upon the wire, being careful not to pull too hard for fear of causing the wire to travel faster than the tissue becomes thoroughly sealed by a burnt crust.

Having made the central incision, a lateral incision made in the same way, divides the anterior pillar including the palato-glossus muscle. A third incision separates the half tongue from the floor of the mouth ; this process may require additional incisions to make the separation satisfactory. Having proceeded thus far, the loop can be slipped over the half tongue which is ready to be amputated; this is gradually accomplished by traction upon the heated wire. At the conclusion of the operation, the surface is dry and of a clear gray color. The patient on waking from his ethereal sleep complains of very little pain. In the experience of the employment of this agent in other regions. very little pain has been complained of, and when employed in the waking state, less pain is complained of than under the employment of knife or scissors.

Case 1.—Dr. C. C., aged about 45, having been in bad health for many years and a slave to tobacco, without syphilitic taint, unless inherited, observed last April a sore on the left side of the tongue opposite the molars. There is now a fissure a quarter of an inch deep on the left edge of the tongue, which is three times its natural thickness. The appearance of degeneration extends back to the anterior pillar, and forward nearly to the point of the tongue. A tumor under the tongue embraces the sublingual gland, which is indurated, and between the finger of one hand in the mouth and the finger of the other under the jaw, the tumor has the feeling of a distinct body. The appearance and the subsequent microscopic examination by Dr. Black, marked the disease of an epithelioma. A profuse salivation kept the patient spitting, and owing to the pain of mastication, liquid food had been subsisted upon for several months.

September 28th, 1875.—The bowels having been freely moved, and five grains of quinine adminstered, the patient at 9 A. M., took 20 drops of nitrite of amyl in an ounce of whiskey, and proceeded to inhale ether. The method of proceeding was that already described, six incisions or distinct applications of the wire loop were made. The sublingual gland was extirpated after the tongue was amputated, and the surface of the xcavation was still further scorched by the wire loop laid on the surface. The duration of the operation, from the commencement of etherization was 65 minutes; no blood was lost except what was occasioned by the vulsellum employ ed to hold the tongue, previous to the adjustment of the wire. The patient went home a distance of ninety miles, the second day, this was a hazardous proceeding, on account of the danger of hemorrhage, on the detachments of the crusts which occurred on the third, fourth, and fifth days; no hemorrhage appeared. There was some difficulty in swallowing for a few hours after the operation, but after once starting there was no further difficulty.

October 26th, the twenty-eighth day from the operation, the patient returned with a growth half an inch in transverse, and three quarters of an inch in antero-posterior diameter, on the inner side of the jaw, and along side of the edge of the tongue. The cicatrization is complete, and the tongue is much impaired in its movements, but not drawn to the left side. The patient being under ether, a thin slice embracing the diseased growth, was removed by the wire loop, after which a deeper burning was effected by a short heavy loop laid upon the surface; swallowing was not impeded by this proceeding. He walked about the next day, and the following day he went home. Under date of November 10th, he wrote: "To day being the fifteenth since the operation, I thought best to report nothing special has happened—no bleeding, sloughing all over in ten days Surface so far soft and showing no sign of hardness or tumor, appetite good, and sleep well, milk, eggs, and beef-tea, my diet—am encouraged so far; as before the tumor developed in granulating process, which is not the case now."

Case 2.—J. W. M., of Samar, Barton Co., Mo., aged 60, was sent to me by my friend Dr. Gregory. There is an ulcerative excavation along the left border of the tongue, tending back to the entrance of the palato-glossus muscles. The sublingual tissue is very little involved; the disease dates from last December. The teeth were drawn about the middle of May with the hope of benefiting the disease. In 1857, or eighteen years ago, there was an ulcer in the right angle of the lips, attributed to smoking, which healed up under the use of arsenic; he has taken arsenic several times for various afflictions, and he is now taking iodide of arsenic by the advice of Dr. Gregory. There is no increased flow of saliva; from the appearance and history of the disease, and subsequent examination by the microscope, the disease is considered an epithelial degeneration, nucleated cells being found in the tissues beyond the encroachment of ulceration.

October 20th, 1875, having taken a cathartic the day before, followed by quinine in the morning, galvano-cautery was commenced at 9 A. M. The etherization was proceded by twenty drops of amyl nitrite in a little syrup and whiskey; the proceeding was the same as that already described, but lasted longer in consequence of the want of activity in the battery, the fluid in which was not entirely new ; this is an admonition never to commence an important operation by galvano cautery, without having the fluid which is to afford the current absolutely new. There was no hemorrhage and the portion removed weighed 540 grains, or 9 drachms. 21st, feels well, and is able to swallow liquids without difficulty ; 22nd, the crusts are separating and the breath fœtid, the smell in his room is neutralized by hanging upon the gas-pendant a sheet wet with a solution of carbolic acid ; 23rd, walks up street ; 24th, bleeding last night and again this morning ; stopped spontaneously. The third and fourth bleedings occurred during the day ; the galvano-cautery applied ; 25th, the fifth bleeding occurred this morning, a more thorough application of the galvano-cautery made a final arrest of the bleedings ; 26th, walks about, subsists chiefly upon milk; 30th, went home.

November 19th, his son writes: "Father is about the same as when he reached home; the side of his face is swollen and he can hardly swallow; he complains of a weakness and soreness in his limbs."

The above cases warrant the conclusion that any portion of the tongue can be removed without fear of primary hemorrhage ; it also affords the best means of combatting subsequent hemorrhage. The galvano-cautery may be imployed in dental surgery, with the most satisfactory advantage, to sever diseased growths from contiguous healthy tissues, to cauterize sinuses, abscesses of the gums, etc., amputation of such parts as the tongue, cervix-uteri, and other parts in the oral cavity, which can not be easily reached by the knife, and to control the hemorrhage from vessels that cannot be conveniently ligatured, important use of this agent may be made in its application for cauterizing nerves of the teeth when exposed, treating alveolar abscess, also, for obtunding sensitive dentine, and thus materially facilitate the after operation of filling the teeth. With a small galvano-cautery battery, cauterizing points, knives, etc., made for me by the Galvano Faradic Manufacturing Company of this city, I have for the past three years pursued a mode of practice, with the most important and satisfactory results.

